# Integrated treatment of first episode psychosis with online training (e-learning): study protocol for a randomised controlled trial

**DOI:** 10.1186/1745-6215-15-416

**Published:** 2014-10-27

**Authors:** Sara Barbeito, Patricia Vega, Sonia Ruiz de Azua, Vicent Balanza- Martinez, Francesc Colom, Esther Lorente, Ana Luengo, Ester Cerrillo, José Manuel Crespo, Ana González Pinto

**Affiliations:** Biomedical Research Centre in Mental Health Net (CIBERSAM), University Hospital of Álava (Santiago Apóstol), Osakidetza, Olaguibel Street, Vitoria, Spain; Basque Country University, Sarriena, s/n, 48940 Leioa, Bizkaia, Spain; University Hospital Doctor Peset, FISABIO, Valencia, Spain; Section of Psychiatry, Department of Medicine, University of Valencia Medical School, CIBERSAM, Valencia, Spain; Barcelona Bipolar Disorders Unit, IDIBAPS-CIBERSAM, Institute of Neurosciences, Hospital Clinic, Villarroel 170, Barcelona, 08036 Spain; Outpatient of Clinical University Hospital of Valencia, CIBERSAM, Valencia, Spain; Inpatient Psychiatric Unit of the Clinical Hospital at Home of Valencia, Valencia, Spain; Neuroscience Group, Bellvitge Biomedical Research Institute (IDIBELL), Barcelona, Spain; Mood Disorders Clinical and Research Units, Psychiatry Department, Bellvitge University Hospital, L’Hospitalet de Llobregat, Cibersam, Barcelona, Spain

## Abstract

**Background:**

The integrated treatment of first episode psychosis has been shown to improve functionality and negative symptoms in previous studies. In this paper, we describe a study of integrated treatment (individual psychoeducation complementary to pharmacotherapy) versus treatment as usual, comparing results at baseline with those at 6-month re-assessment (at the end of the study) for these patients, and online training of professionals to provide this complementary treatment, with the following objectives: 1) to compare the efficacy of individual psychoeducation as add-on treatment versus treatment as usual in improving psychotic and mood symptoms; 2) to compare adherence to medication, functioning, insight, social response, quality of life, and brain-derived neurotrophic factor, between both groups; and 3) to analyse the efficacy of online training of psychotherapists.

**Methods/design:**

This is a single-blind randomised clinical trial including patients with first episode psychosis from hospitals across Spain, randomly assigned to either a control group with pharmacotherapy and regular sessions with their psychiatrist (treatment as usual) or an intervention group with integrated care including treatment as usual plus a psychoeducational intervention (14 sessions). Training for professionals involved at each participating centre was provided by the coordinating centre (University Hospital of Álava) through video conferences. Patients are evaluated with an extensive battery of tests assessing clinical and sociodemographic characteristics (Positive and Negative Syndrome Scale, State-Trait Anxiety Inventory, Liebowitz Social Anxiety Scale, Hamilton Rating Scale for Depression, Scale to Assess Unawareness of Mental Disorders, Strauss and Carpenter Prognostic Scale, Global Assessment of Functioning Scale, Morisky Green Adherence Scale, Functioning Assessment Short Test, World Health Organization Quality of Life instrument WHOQOL-BREF (an abbreviated version of the WHOQOL-100), and EuroQoL questionnaire), and brain-derived neurotrophic factor levels are measured in peripheral blood at baseline and at 6 months. The statistical analysis, including bivariate analysis, linear and logistic regression models, will be performed using SPSS.

**Discussion:**

This is an innovative study that includes the assessment of an integrated intervention for patients with first episode psychosis provided by professionals who are trained online, potentially making it possible to offer the intervention to more patients.

**Trial registration:**

NCT01783457 clinical trials.gov. Date of registration in primary registry 23 January 2013.

## Background

The definitions of the words psychotic and psychosis [1] refer to the occurrence of delusions and hallucinations, without specifying their nature in terms of the underlying disease or mental illness. The incidence of psychosis falls with age and is twice as high in men as in women [2]. Around 20% of patients with a first psychotic episode will have no further episodes [3]. This means, however, that symptoms do recur in the great majority of patients.

A recent meta-analysis indicated that the factors that best predict the occurrence of relapses are failure to adhere to treatment, persistent substance abuse disorders, negative comments from caregivers and poor premorbid adjustment [4]. With regards to treatment, previous studies have demonstrated that individuals who receive early intervention including guidance on how to improve their adherence to treatment, insight into their illness and self-management and how to minimise substance use have a better course and, hence, these factors are associated with a better prognosis [5–10]. Indeed, it has been observed that delays in the treatment of psychosis tend to be associated with slower recovery, greater suffering, a higher level of comorbidity and greater deterioration in family and social relationships [11]. Further, the critical period of the disease (the first 5 years) is a relatively reliable indicator of the long-term course of the disease [12, 13]. Overall, it seems that interventions carried out as early as possible improve prognosis and quality of life in these patients.

Regarding the types of treatment for patients with first episode psychosis, a review concluded that early psychosocial interventions adjunctive to pharmacotherapy may contribute to patient symptomatic and functional recovery [14]. Various types of treatment have shown efficacy in these patients. For instance, some authors recommend cognitive behavioural therapy during the acute stage of the disease or during remission [15]. However, systematic reviews have found that integrated treatment (including biological, psychological and social elements) is the approach that most significantly reduces the rates of transition to psychosis and negative symptomatology compared to treatment as usual. Further, integrated treatment has proven efficacy in various areas, being associated with less psychotic symptomatology at 2 years follow-up, fewer negative symptoms, better global functioning, lower rates of substance abuse, lower doses of the second-generation antipsychotic medications and fewer days of hospitalization [16–19]. A clinical trial also found that this type of treatment produces good results in the first stages [20]. Nevertheless, there is a need for more randomised controlled clinical trials to assess the effectiveness of these interventions [14].

On the other hand, it has been shown that disciplines, such as cognitive behavioural therapy, that have a shortage of formally trained clinicians could benefit from online learning [21]. In particular, a European project has demonstrated the effectiveness of teaching integrated treatment through the web [22]. New forms of transmission of these skills could be an important way for clinicians to gain access to specialist knowledge, and facilitate access to training for more clinicians.

This study hopes to shed light on these issues, with an improved methodology; it is a pragmatic trial - that is, there is no selection of a specific type of patients - and the aim is to assess the effectiveness of individual psychoeducation as well as the remote training of therapists.

The primary objective of this study is to assess the efficacy of a psychoeducation programme in improving the symptomatology of patients; namely the positive, negative, and general symptoms of psychosis, measured using the Positive and Negative Syndrome Scale (PANSS), State-Trait Anxiety Inventory (STAI), and Hamilton Rating Scale for Depression (HRSD).

The secondary outcomes of interest are patient functioning (measured using the Strauss and Carpenter Prognostic Scale, Global Assessment of Functioning (GAF) scale, Functioning Assessment Short Test (FAST)), adherence (Morisky Green Medication Adherence Scale), insight (Scale to Assess Unawareness of Mental Disorders (SUMD)), social response (Liebowitz Social Anxiety Scale (LSAS)), quality of life (World Health Organization Quality of Life instrument WHOQOL-BREF (an abbreviated version of the WHOQOL-100) and the EuroQoL questionnaire), and brain-derived neurotrophic factor (BDNF) level.

We also want to analyse the efficacy of online training of psychotherapists.

In this paper, we describe in detail the study design, patient selection and evaluation, the programme of psychoeducation under study, and training of professionals. It is an intervention study of integrated treatment for patients with first episode psychosis, with the innovation that the participating therapists will receive remote training.

## Methods

### Design

We registered this single-blind randomised clinical trial in 2013 (identifier NCT01783457). Patients with a first episode of psychosis between 2010 and 2014 are to be included and randomly assigned to one of two treatment groups: the control group, receiving pharmacotherapy together with regular sessions with a psychiatrist (treatment as usual), and the intervention group receiving integrated treatment, namely treatment as usual plus a psychoeducational intervention (12 main sessions and 2 booster sessions). A first psychotic episode was defined as the first time a patient displayed positive psychotic symptoms of delusions and/or hallucinations.

We are recruiting patients from five psychiatric centres (University Hospital of Álava, Clinic Hospital of Barcelona, Clinical Foundation of Valencia, University Hospital of Bellvitge, and University of Valencia) that agreed to take part in the e-learning project. In all centres, the clinicians recruit subjects who show psychotic symptoms for the first time, and who meet the inclusion criteria (listed below). To obtain 90% power to detect differences in the means at the 5% level of significance, we have estimated that we need to enrol 130 patients in each group (study group and controls) (alpha = 0.05; beta = 0.10; n = 260).

The treatment is being supervised by one clinician (PV) trained by a highly experienced clinician at the University Hospital of Álava (SB), while the evaluations in all centres are conducted by other clinicians blind to patient allocation.

#### Inclusion criteria

For patients to be included, they must have experienced a first episode of psychosis. All those included must have met *Diagnostic and Statistical Manual of Mental Disorders* 4^th^ edition text revised (DSM-IV-TR) criteria for one of the following within the previous 5 years: schizophreniform disorder, schizoaffective disorder, brief psychotic disorder, delusional disorder, non-specified psychotic disorder, bipolar disorder with psychotic symptoms, or major depressive disorder with psychotic symptoms. In addition to giving informed consent, they must also be between 18 and 45 years old and speak good Spanish.

#### Exclusion criteria

Subjects with mental retardation, organic brain disorders or the presence of any comorbid condition that could hinder communication, or drug abuse as a primary diagnosis, were excluded.

#### Ethical considerations

The study is conducted in compliance with local regulations and internationally established principles of the Declaration of Helsinki (64^th^ World Medical Association General Assemble, Fortaleza, Brazil, 2013). The study and protocol were approved by the Clinical Research Ethics Committee of each participating health centre (University Hospital of Álava: HS/PI2010009: Clinic Hospital of Barcelona: 2010/5890, Clinical Foundation of Valencia; 2010/2904, University Hospital of Bellvitge: PR079/10, and University of Valencia: 2010/2904). Before inclusion, all patients are required to sign an informed consent form that includes a specific section covering genetic testing (for the BDNF analysis, see below).

#### Randomisation

All individuals with first episode psychosis that meet the selection criteria are randomised with random allocation software. For this purpose, all the groups from the participating centres send the initials of each patient recruited to researchers at the coordinating centre who specify the allocation of the patient (to the control or intervention group); the person in charge of the assessments is blind to this process.

#### Pharmacotherapy

For all participants, the pharmacological treatment used is that which patients have been prescribed by their psychiatrists, independent of their participation in the study.

### Patient assessment

Data are collected on the wide range of clinical and demographic variables detailed below and are entered on a data collection form. All the patients are assessed at baseline and have been or will be assessed at 6 months after inclusion in the study, which corresponds to the end of the psychoeducational programme in the case of the intervention group.

#### Demographic data

Data are collected on age, sex, level of education, living arrangements and employment status.

#### Symptomatology

Participants’ symptoms are assessed using the PANSS [23]. This instrument is composed of 30 items that assess positive and negative symptoms as well as the general psychopathology of the disorder. Each item is measured using a 7-point Likert scale reflecting the severity of symptoms, from 1 (none at all) to 7 (extreme symptoms). This scale has been validated in Castilian Spanish [24].

Trait and state anxiety are measured using the STAI [25]. This instrument has two parts: one assesses the anxiety experienced by patients in the previous week (state) using 20 items with four response options; while other part, also composed of 20 items with four response options, assesses their usual reactions to certain situations (trait anxiety). The STAI was adapted from the version described in a Spanish paper by Bermúdez in 1978 [26]; across all these items the mean and the reliability (Cronbach’s alpha and test-retest correlation) are similar to those of the original version [27].

Patients’ social anxiety is assessed using the LSAS [28], and its psychometric properties have been found to be satisfactory in the Spanish population [29]. Lastly, mood is evaluated using the HRSD [30], which has also been validated in Castilian Spanish [31].

In addition, in relation to symptoms, we record information on the number of relapses and hospital admissions (psychotic episodes) and the dates on which they occur for 2 years follow-up, the psychopharmacotherapy administered, family history of mental health problems, and record of substance use both before and after the intervention.

#### Patient awareness of their disease

Patient insight into their illness is measured using SUMD [32]. This scale provides broad assessment of patient thoughts and beliefs regarding their disease and medication. The Spanish adaptation of the SUMD is equivalent to the original version, and it has a similar reliability and external validity [33].

#### Prognosis

Prognosis is explored using the Strauss and Carpenter Prognostic Scale [34–36], an instrument that assesses the best status of the patient in the previous year in four areas: hospitalisations, work, social activity, and global functioning and, on the other hand, symptoms considering the previous month only. It is an interview-administered instrument with Likert-type response options ranging from 0 to 4, higher scores indicating a better prognosis. It has been validated in Castilian Spanish for schizophrenia [37].

#### Functioning

The overall function of patients is rated using a Spanish version of the GAF scale. This scale is recommended for Axis V in the multiaxial system of DSM-IV-TR [38] and is based on the opinion of clinicians regarding the level of general activity and functioning of patients. It has been validated in individuals with severe mental disorders [39].

Functional status is also assessed using FAST [40]. This is an interview-administered scale that assesses a patient’s functioning over the previous 2 weeks in six areas: autonomy, occupational functioning, cognitive functioning, financial issues, interpersonal relationships and leisure time. The FAST showed strong psychometric properties in Spanish individuals with first episode psychosis [41].

#### Treatment adherence

Adherence is estimated using the Morisky Medication Adherence Scale [42]. This instrument has been validated for the Spanish population [43]. It has four questions and assesses the attitudes of the patients towards their treatment. Patients with a score of 4 were considered to have ‘good’ adherence, while those with a score between 0 and 3 were classified as having ‘bad’ adherence.

#### Quality of life

The quality of life is measured using two instruments. First, the World Health Organization Quality of Life WHOQOL-BREF questionnaire [44] was used; this short version being composed of 26 items exploring four dimensions: physical health, psychological health, social relationships and the environment. Spanish field trials have confirmed that this questionnaire has adequate psychometric properties [45].

Second, the EuroQoL questionnaire [46] was used. The main part of this measures five dimensions (EQ-5D): mobility, self-care, usual activities, pain/discomfort and anxiety/depression. It also contains a visual analogue scale (EQ-VAS) represented by a vertical line on which subjects rate their self-perceived health status from 0 (the worst) to 100 (the best imaginable health status). The questionnaire is considered a simple, valid and practical measure and the Spanish version has been validated [47].

All the instruments used have demonstrated adequate psychometric properties and have been used in many other studies. Interviews are conducted by clinicians who have good inter-rater reliability for the scales used (PANSS, kappa = 0.80; STAI, kappa = 0.77; GAF, kappa = 0.95; LSAS, kappa = 0.83; HDRS-21, kappa 0.79; SUMD, Kappa = 0.81; Strauss–Carpenter, kappa = 0. 79; Morisky Medication Adherence Scale, Kappa = 0.94, FAST, Kappa = 0.88, WHOQOL-BREF, Kappa = 0.81 and Euroquol, Kappa = 0.78).

#### Biochemical analysis

To investigate BDNF in peripheral blood, blood samples are collected in the morning (on two occasions, like the other assessments, once at baseline and once at 6 months after inclusion). Plasma BDNF levels are measured using a BDNF Sandwich enzyme-linked immunosorbent assay kit (CYT306; EMD Millipore Corporation, Billerica, MA, USA), according to the manufacturer’s instructions. Blood cells are immediately separated from the plasma and processed. Standard curves are constructed using plasma duplicates. The absorption at 450 nm is measured with a Synergy HT microplate reader (BioTek Instruments, Winooski, VT, USA).

### Online training

The coordinating group carried out the training of the therapists in the participating centres, via teleconferencing (WebEx Training Center). The course consisted of 12 modules and 2 booster sessions, corresponding to the 14 sessions of the psychoeducational programme, and all the participating groups received the same theoretical content. Once all the materials had been provided and explained, participating groups were asked to perform a task with certain objectives, each group having to complete this before starting the following module.

In addition, all the therapists participating in the online training had to prepare a case study with theoretical and practical elements and correctly complete a task under the supervision of a psychotherapist with extensive experience in the field of psychoeducation, before they themselves carried out the psychoeducational intervention with the patients. As well as providing written support material, we attempted to ensure the correct integration of knowledge presented by giving feedback, correcting answers participants gave during sessions, and correcting hypothetical cases presented. At the end of each module, all the therapists were required to complete a form recording the learning objectives covered in the module and testing their knowledge of theoretical and practical aspects of the psychoeducation programme.

### Intervention programme

#### Integrated treatment

The psychoeducational programme has 14 1-hour sessions (12 main sessions and 2 booster sessions), fortnightly, for a period of 6 months, focused on improving patient insight into their illness, treatment adherence, prodromal identification, early intervention to prevent relapses, healthy lifestyles, techniques for managing anxiety, social skills and problem solving.

The programme includes the following sessions:

1. What is a first episode of psychosis?
2. The challenge and importance of insight into vulnerability
3. Symptom recognition
4. Prevention of relapses: protective and risk factors
5. What can I do if I note the symptoms emerging again?
6. Treatment adherence
7. Healthy lifestyles: sleep and sexuality
8. Healthy lifestyles: substance use
9. Anxiety management techniques (I)
10. Anxiety management techniques (II)
11. Social skills: assertiveness techniques
12. Problem-solving techniques

We have checked the completeness of the training with respect to the administration of the sessions both in terms of content and their structure. The psychoeducational sessions are run with the same interval between sessions in all the centres. Further, the therapists give the psychoeducational intervention to all patients in the same way and provide the same information, enhancing the reliability of the study.

If patients report any personal, social, work-related or family problems during the sessions, they are to be helped using cognitive behavioural techniques (psychotherapeutic intervention). Lastly, if patients have any question regarding what they are taught during the sessions, or about their condition, they can call a telephone helpline in working hours (between 08.00 and 15.00 from Monday to Friday). The aim of this service is to facilitate patient access to information and address any questions they might have.

#### Treatment as usual

Treatment as usual refers to the treatment that is routinely provided to patients with first episode psychosis on the Spanish National Health Service. It consists of ambulatory psychopharmacotherapy with regular sessions with an assigned psychiatrist, corresponding to a personalised psychiatric approach to their management.

### Data processing

We used Gridsam, the central database of the Spanish research network for mental health (CIBERSAM). This database facilitates the merging of large amounts of data and access to the records.

Comparisons of baseline characteristics of the sample will be made with χ^2^-tests for categorical variables and the Student’s *t*-test or Mann Whitney U test for quantitative variables. Within-group changes in symptomatology and functioning during treatment will be examined using paired *t*-tests.

Univariate analyses of covariance will be conducted to investigate differences between groups. For this, the independent variable is the group assignment (integrated treatment versus control) and the dependent variable is the respective clinical or functional outcome score post-treatment. Recurrence-free curve analysis will be performed using Kaplan–Meyer survival analysis to analyse the relapses in the groups during the follow-up. This analysis will be performed on an intention-to-treat basis, with patients being analysed in the treatment group to which they were randomised.

In addition, a logistic regression model will be used to analyse the association between clinical variables. Finally, we will also build a logistic regression model to explore factors associated with changes in variables during follow-up.

The outcomes will be reported with 95% confidence intervals. All data will be analysed using SPSS (version 21.0; IBM Corp. Released 2012. IBM SPSS Statistics for Windows, Version 21.0. Armonk, NY: IBM Corp.

## Discussion

The project we describe in this paper represents an innovation in our setting given that it uses an online training platform, in which supervision, training and question and answer sessions are carried out remotely, facilitating access by all professionals, and reducing financial and time costs.

On the other hand, the integrated treatment itself is based on strategies that have proven to be effective, and, depending on the results, this project might lead to its adoption in routine practice in our setting. In addition, the close monitoring of first episodes of psychosis associated with the study provides extra data, improving our knowledge and understanding of the situation of these patients and, in turn, allowing treatments to be optimised. Finally, the proposed intervention at the onset of the disease will enable the patients to reap the benefits of early intervention, not only at the pharmacological level, but also in the areas addressed by adjunctive treatments that have proven to be effective [48], but that are not currently in the portfolio of services offered in our health centres.

With regards to the limitations of this study, first of all some variables are measured by self-report and patients could under- or overestimated their symptoms, but patients are also assessed by clinicians. Further, although the scales employed are among the most widely used by clinicians and researchers in this type of patient, results may not be comparable between studies if different instruments are used. Finally, although we have already recruited many patients and recruitment continues to the end of 2014, the power of the study would be increased with a larger sample size and, to assess the longer-term impact of the intervention, a longer monitoring period should be considered (>6 months). On the other hand, this is the first study in our setting assessing this type of therapy and this type of innovative training with a rigorous methodology (structure and content).

## Trial status

We have not completed patient recruitment at the time of submission.

## Abbreviations

BDNF: brain-derived neurotrophic factor
DSM-IV-TR: *Diagnostic and Statistical Manual of Mental Disorders* 4^th^ edition text revised
FAST: Functioning Assessment Short Test
GAF: Global Assessment of Functioning
HRSD: Hamilton Rating Scale for Depression
LSAS: Liebowitz Social Anxiety Scale
PANSS: Positive and Negative Syndrome Scale
STAI: State-Trait Anxiety Inventory
SUMD: Scale to Assess Unawareness of Mental Disorders
WHOQOL-BREF: World Health Organization Quality of Life instrument.
